# The effect of Carbaryl on the pituitary-gonad axis in male rats

**Published:** 2012-09

**Authors:** Esmail Fattahi, Seyed Gholam Ali Jorsaraei, Mossa Gardaneh

**Affiliations:** 1 *Department of Biology, Islamic Azad University, Ayatollah Amoli Branch, Amol, Iran.*; 2 *Fatemeh Zahra Infertility and Reproductive Health Research Center, Babol University of Medical Sciences, Babol, Iran.*; 3 *Department of Molecular Genetics, National Institute of Genetic Engineering and Biotechnology, Tehran, Iran.*

**Keywords:** *Carbaryl*, *Testis*, *LH*, *FSH*, *Spermatogenic cells*

## Abstract

**Background:** Carbaryl is a carbamate insecticide widely used to control pests in agriculture and farm. Carbaryl adversely affect the reproductive endocrine systems in animals.

**Objective:** The aim of this study was to evaluate Carbaryl effects on the pituitary-gonad axis in rats.

**Materials and Methods:** In this experimental - analytical study, 60 adult male rats were divided into four equal groups: control, sham and experimental (1 and 2) groups that received 10 and 30 mg/kg Carbaryl via intraperitoneally injection. The sham group was subjected to intraperitoneally injection with olive oil while the control group did not receive any injection. Animals were sacrificed 35 days after the last treatment. Tissue sections were prepared from testes to investigate possible changes occurring in spermatogenic and Leydig cells. Blood samples were collected in which the levels of testosterone, luteinizing hormones (LH) and follicle stimulating hormone (FSH) were measured.

**Results:** The results showed significant reduction in testes weight (p=0.042) and seminiferous diameters (p<0.001) within the experimental groups compared with control group. Also, the number of germ cells, spermatocyts, spermatids and Leydig cells on the testes of the experimental groups was significantly decreased (p<0.001). Accordingly, significant decline in the testosterone levels (p<0.001) and increase in LH and FSH levels were observed (p<0.05).

**Conclusion:** These results demonstrated that Carbaryl has capacity to exert adverse effects on fertility. Therefore, have to be taken to account in applying Carbaryl for any studies and or commercial use.

## Introduction

Ecosystems, in particular water ecosystems, are constantly contaminated by toxic chemical compounds that originate from industrial, agricultural and domestic sources. Pesticides constitute one of the main sources of toxic chemical compounds (1, 2). Pesticides are used to kill agricultural pests and eradicate diseases and Carbaryl is considered one of the strongest pesticides available in agriculture (3). 

Carbaryl with its chemical name of 1-naftol-N-methyl carbamate is a member of carbamates derived from carbamic acid. Carbaryl inhibits choline esterase (4, 5). The range of damages pesticides inflict on our body organs and tissues depends on contact procedures, dose of the pesticide, biological changes and accumulation of metabolites (6). 

Sub-lethal doses of Carbaryl leave behind various biochemical effects and damage different tissues and body organs specially the reproductive system (7). Carbaryl and its metabolites can adversely affect living organisms and long-term effects of Carbaryl results in a reduced rate of movements in fisheries. Carbaryl can, in the long term, reduce sperm movement and make changes in spermatogenesis (8, 9). Pant and co-workers report that Carbaryl does not change testes weight, epididim and related sexual organs. But they observed histopathological changes in testes tissue (10). 

Examples of Carbaryl adverse effects are reduction in sperm number and sperm movement and increased rate of sperm malformations. Reduced rate of mating and reduced fetus size beside its increased mortality rate in rats exposed to Carbaryl have been detected (11, 12). Reduction in oocyte size and numbers, as well as oocyte malformation at various stages and increased number of atritic follicles are other examples of Carbaryl-mediated toxicity on the ovary (13). Several studies have shown that Carbaryl causes genetic damages within live cells and so is considered a mutagen in vitro and in vivo (14). 

Carbaryl and other toxins can destroy the endocrine systems leading to reduced or increased levels of hormones. For example, estradiol, testosterone, and progesterone levels are reduced due to Carbaryl toxicity (15). Carbaryl inhibits DNA synthesis and reduces RNA levels, changes that ultimately result in reduced levels of protein expression and so limited growth (16). In the present study, we examined the impact of Carbaryl on rat testes.

## Materials and methods

**Animals**

Sixty 10-12 week-old male Wistar rats each weighing 150-200 gr were purchased from Pasteur Institute of Iran and equally divided to 4 groups: experimental groups 1 and 2, control and sham. The animals were maintained in standard cages at 25±2^o^C under conditions of 12 hours light and 12 hours dark, with access to powdered diet and to deionized water. Work with these animals was carried out according to the regulations set by the ethical committee in the research deputy of Babol University of Medical Sciences.

**Chemicals**

Carbaryl, a commercial formulation (85% active ingredient), was obtained from Trading Company of China. Carbaryl was dissolved in olive oil and injected intraperitoneally in sub lethal doses.

**Carbaryl injection**

Rats within experimental groups 1 and 2 received intraperitoneal (i.p.) injection, respectively, of 10 mg/kg and 30 mg/kg Carbaryl (17). The sham group was injected with olive oil but the control group was left without any injection. All animals were maintained under optimum conditions for 35 days and sacrificed for sampling afterwards. 

**Tissue sample preparation**

To examine testes tissue and Leydig cells, the testes were extracted and fixed in 10% formalin solution. The tissues were serial sectioned with 5 µ thickness and stained with hematoxylin and eosin (H&E). Then various cell types including Leydig cells were counted using the eyepiece graticule. The diameters of seminiferous tubules and testes were measured, respectively, using the eye-piece and micrometer.

**Measurement of hormone levels**

In order to measure the level of testosterone, LH and FSH, blood samples were collected from the animals and subjected to centrifugation at 3000 rpm for 15 min to separate their serum. Then hormonal levels were measured by radioimmunoassay kit (USCN. Co).

**Statistical analysis**

Data in the figures are represented as the mean±SEM of 3 or more separate experiments carried out in triplicate. We analyzed our data using SPSS version 16 and applied one-way ANOVA and t-test. P<0.05 were considered statistically significant, and p<0.01 as highly significant.

## Results

**Changes in the reproductive tract**

This parameter was found to be reduced in experimental groups 1 and 2 compared to control and sham groups. This reduction was statistically significant. Sublethal dose of Carbaryl in experimental groups markedly reduced the diameter (p<0.001) and weight (p=0.042) of testes. Further examination of animal groups showed that significant reduction has occurred in the number of germ cells (p<0.001) and spermatocyts in our experimental groups (p<0.001). 

Comparison between spermatids among various animal groups indicated that their number has significantly been reduced in experimental groups compared to sham and control groups (p<0.001). This reduction was more visible in experimental group 2. Microscopic examination of testes tissue showed reduced number of Leydig cells in both experimental groups compared to sham and controls (p<0.001). Also sections prepared from testes showed that the diameter of seminiferous tubules has been reduced in the experimental groups compared to sham and control groups and this reduction was significant (p<0.001) (Table I, Figure 2).

**Sex-related hormones**

**Figure 1:**
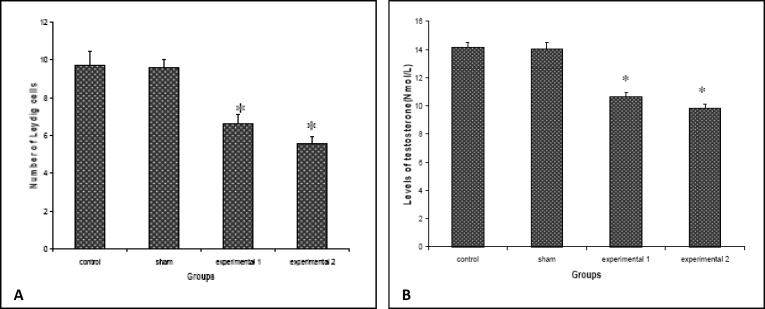
The effects of Carbaryl injection on the number of Leydig cells (A) and levels of testosterone (B) in different animal groups (* p<0.001).

The levels of testosterone in blood serum indicated that its average levels have been significantly reduced in experimental groups compared to sham and control groups (p<0.001). Reduction in testosterone levels was more pronounced in experimental group 2 which had received three times more toxin (Figure 1). In contrast, the level of LH within the serum of the experimental groups was found to be increased compared to sham and control groups, and this increase was statistically significant (p<0.05). 

Also measurement of FSH indicated that its average levels has increased significantly (p<0.05). However, we did not detect any significant differences between sham and control groups (Table II).

**Table I:** The effect of Carbaryl on testes tissue (Mean±SD) in different animal groups

**Rats exposure**	**Control**	**Sham**	**Experiment 1** **(10mg/kg)**	**Experiment 2** **(30mg/kg)**	**p-value**
Testis weight (gr)	7.32±0.06	7.28±0.07	6.9±0.17	6.68±0.13	0.042
Diameter of testes (mm)	8.56±0.11	7.49±0.08	7.02±0.04	6.53±0.049	<0.001
Germ cells	8.27±0.42	8.2±0.34	6.45±0.399	6.12±0.22	<0.001
Spermatocyts	32.3±1.13	31.95±1.23	28.32±0.93	27.97±1.2	<0.001
Spermatids	10.14±0.21	10.01±0.14	8.25±0.24	7.82±0.2	<0.001
Diameter of seminiferous tubules (μm)	81.12±0.78	80.89±0.9	68.45±0.62	67.59±0.81	<0.001

**Table II:** The effects of Carbaryl on hormones levels (Mean±SD) in different animal groups

**Rats exposure**	**Control**	**Sham**	**Experiment 1 (10mg/kg)**	**Experiment 2 (30mg/kg)**	**p-value**
LH (U/L)	5.12±0.51	5.16±0.49	6.3±0.516	6.98±0.51	<0.05
FSH (U/L)	3.25±0.51	3.3±0.49	4.63±0.34	4.96±53	<0.05

**Figure 2:**
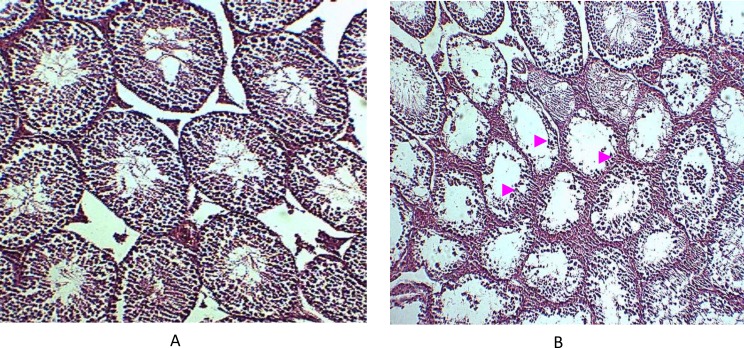
Light micrograph of cross section from rats testes in control (A) and Carbaryl (B) groups, staining with H&E (40X). Treated rats show decrease in the number of spermatogenic lines in the seminiferous tubules (arrows).

## Discussion

Numerous studies indicate that a diverse range of contaminants, chemicals and gases can affect the reproduction process in humans and animals, leaving behind damages that, in some cases, cannot be compensated (4). The results of our current study indicate that Carbaryl causes reduction in spermatogenic, Leydig cells, diameter and weight of testes and seminiferous tubules. These results further show that Carbaryl results in reduced levels of testosterone and increased levels of LH and FSH in Wistar rats. 

Carbamates damage the reproductive tract by changing activities of anti-oxidant enzymes and so accelerate free radical generation and induce peroxidation and destruction of intracellular macromolecules (18). These are the case with Carbaryl that directly affects reproductive cells and tissues, causes damages to testes conformation and various cell types during spermatogenesis, and changes levels of reproductive hormones (14). 

The average number of sperms in men has been reduced in the last 50 years due, in part, to the use of environmental chemical compounds specially toxins in agriculture. Toxins that humans are daily exposed to due to their occupation can disturb sperm production by affecting testes cells directly or hormone regulation indirectly during spermatogenesis. These changes appear as reduced sperm levels, increased production of defective sperms and disturbed levels of androgen production (19).

Destructive functions of toxins depend on dose, contact and adsorbing procedures, mechanism of action, type of tissue and organ, produced metabolites and their stability in the body (20). Carbaryl causes chromosomal changes, chromatid exchanges, mutations and DNA damages. It inhibits DNA synthesis and reduces RNA levels leading to reduced levels of protein synthesis and processing. These changes result in limited growth (16, 21). 

Reduction in testes weight indicates that Carbaryl may have damaged testes tissue and that reduced cell types and seminiferous tubules diameter most likely cause in testes atrophy. Carbaryl decreases the enzymatic activity of alkaline phosphatase that plays an important role in spermatogenesis (22). Further studies indicate that Carbaryl has destructive effects on reproductive systems of various species, changes that range from reduced sperm number and slowed sperm movement as well as sperm defection. Reduced rate of mating, decreased size of the embryo and increased rate of fetal death have been observed in rats following their contact with Carbaryl (23). 

Carbamate pesticides such as carbaryl disturb mitochondrial function that results in reduced ATP production. They also block metabolism of proteins and nucleic acids causing cell death. Carbaryl adversely affects sperms and germinal cells by inhibiting growth and inducing cell death (24, 25). Decrease of germinal cells might be the cause of decreases in the number of spermatocyts and spermatids, which would eventually result in a decrease of sperm. The diameter of seminiferous tubules have showed a significantly reduction after prolonged Carbaryl exposure, which may be due to decreased of spematoginc cells and or may be occurring due to atrophy.

The results of our study revealed the reduced levels of testosterone and increased levels of LH and FSH in the blood. Toxicologic effects exerted by toxins such as carbaryl and chloropyriphos occur probably by affecting the endocrine system and or changes in hormonal function (22, 26). The average level of testosterone detected in our experiments showed significant reduction in the experimental groups compared to sham and control groups. 

Our data indicate that Leydig cells responsible for secreting testosterone have been reduced in treated animals. Therefore, reduced levels of serum testosterone were not unexpected following reduction in the number of Leydig cells. Testosterone is required for production of sperms. Therefore, a normal level of testosterone plays an important role in spermatogenesis and fertility. Decline of testosterone levels might to affecting in spermatogenesis and fertility. Our data are compatible with those previously reported by others (27, 28). 

Carbaryl and other carbamate toxins affect steroid hormones by accelerating their catabolism, inhibiting steroid acute regulatory (STAR) protein, disturbing the endocrine system, directly affecting testes tissue and hormone-producing cells and finally changing the cAMP signaling pathway (15, 29). Non-systemic pesticides such as organophosphores, pyrothyroids and carbamates reduce blood levels of estradiol and prolactin (30). 

In our study, the levels of FSH and LH increased upon consecutive injections of Carbaryl. It is noteworthy that STAR present in Leydig cells is induced by LH and some carbamates inhibit signaling pathways involved in LH and prolactin production (31, 32). This is while other carbamates such as methomyl increase LH and FSH levels without inducing prolactin and thyroid-stimulating hormone (33). 

Therefore, Carbaryl that increased hormone levels in our study fits within this latest group of carbamates since it induced LH levels via feedback inhibition. LH plays an important role in the initiation and continuation of inhibition of spermatogenesis in its various stages and so LH increase can reduce cell number of testes tissue. Therefore, we can claim that beside LH increase leading to germinal and Leydig cell numbers, it is possible that toxins such as Carbaryl introduce a secondary infertility by exerting conformational changes in the testes and destruction of reproductive cells

## Conclusion

Pesticides can damage reproductive systems in species and these damages are complicated via complicated mechanisms. Hormonal disturbance might be one of these mechanisms and, considering the role of hormonal homeostasis in reproduction, we can conclude that these toxins exert their adverse effects on the reproduction process via disturbing hormonal homeostasis. 

Another pathway for toxicity could be a direct effect on testes and reduction in spermatogonia and Leydig cells. Due to use of chemical toxins in farms and considering the data we obtained in this study, it is proposed that the application of toxin type, amount and time need to be carried out with careful planning and thoughtful management.
